# Phenotypic and functional characterisation of CCR7^+ ^and CCR7^- ^CD4^+ ^memory T cells homing to the joints in juvenile idiopathic arthritis

**DOI:** 10.1186/ar1485

**Published:** 2005-01-12

**Authors:** Marco Gattorno, Ignazia Prigione, Fabio Morandi, Andrea Gregorio, Sabrina Chiesa, Francesca Ferlito, Anna Favre, Antonio Uccelli, Claudio Gambini, Alberto Martini, Vito Pistoia

**Affiliations:** 1 II Division of Pediatrics, University of Genoa, Genoa, Italy; 2 Laboratory of Oncology, 'G. Gaslini' Institute for Children, Genoa, Italy; 3 Department of Surgery, 'G. Gaslini' Institute for Children, Genoa, Italy; 4 Neuroimmunology Unit, Department of Neurosciences, Ophthalmology and Genetics and Centre of Excellence for Biomedical Research, University of Genoa, Genoa, Italy; 5 Department of Pathology, 'G. Gaslini' Institute for Children, Genoa, Italy

**Keywords:** chemokines, memory T lymphocytes, juvenile idiopathic arthritis

## Abstract

The aim of the study was to characterise CCR7^+ ^and CCR7^- ^memory T cells infiltrating the inflamed joints of patients with juvenile idiopathic arthritis (JIA) and to investigate the functional and anatomical heterogeneity of these cell subsets in relation to the expression of the inflammatory chemokine receptors CXCR3 and CCR5. Memory T cells freshly isolated from the peripheral blood and synovial fluid (SF) of 25 patients with JIA were tested for the expression of CCR7, CCR5, CXCR3 and interferon-γ by flow cytometry. The chemotactic activity of CD4 SF memory T cells from eight patients with JIA to inflammatory (CXCL11 and CCL3) and homeostatic (CCL19, CCL21) chemokines was also evaluated. Paired serum and SF samples from 28 patients with JIA were tested for CCL21 concentrations. CCR7, CXCR3, CCR5 and CCL21 expression in synovial tissue from six patients with JIA was investigated by immunohistochemistry. Enrichment of CD4^+^, CCR7^- ^memory T cells was demonstrated in SF in comparison with paired blood from patients with JIA. SF CD4^+^CCR7^- ^memory T cells were enriched for CCR5^+ ^and interferon-γ^+ ^cells, whereas CD4^+^CCR7^+ ^memory T cells showed higher coexpression of CXCR3. Expression of CCL21 was detected in both SF and synovial membranes. SF CD4^+ ^memory T cells displayed significant migration to both inflammatory and homeostatic chemokines. CCR7^+ ^T cells were detected in the synovial tissue in either diffuse perivascular lymphocytic infiltrates or organised lymphoid aggregates. In synovial tissue, a large fraction of CCR7^+ ^cells co-localised with CXCR3, especially inside lymphoid aggregates, whereas CCR5^+ ^cells were enriched in the sublining of the superficial subintima. In conclusion, CCR7 may have a role in the synovial recruitment of memory T cells in JIA, irrespective of the pattern of lymphoid organisation. Moreover, discrete patterns of chemokine receptor expression are detected in the synovial tissue.

## Introduction

Migration and accumulation of memory T cells in the synovium is a critical step in the pathogenesis of chronic arthritides [1-3]. Chemokines are a large family of small secreted proteins (8–15 kDa) that control lymphocyte trafficking in physiological and pathological processes. The evaluation of type and distribution of chemokines and their receptors in the synovium is therefore crucial to an understanding of the mechanisms of synovial T cell recruitment.

From a functional point of view, chemokines can be broadly classified into two groups: inflammatory and homeostatic [4]. The inflammatory chemokines are induced by proinflammatory stimuli and control the migration of leukocytes to the site of inflammation. CCR5 and CXCR3 are classical examples of receptors for inflammatory chemokines [5]. The homeostatic chemokines regulate the basal traffic of lymphocytes and other leukocytes through peripheral lymphoid tissues. CCR7 is an example of a receptor for homeostatic chemokines. CCR7 and its ligands (CCL19 and CCL21) have also been shown to have a pivotal role in the development and maintenance of secondary lymphoid organ microarchitecture [4,5]. Recently, the CCR7 chemokine receptor has been identified as an important marker of memory T cell differentiation. It has been proposed that CCR7^+ ^memory T cells represent a pool of 'central' memory T cells homing to lymph nodes, where they undergo further differentiation into CCR7^- ^memory T cells, which migrate to the peripheral tissues to perform their effector functions [6].

However, this model has been disputed by other investigators [7,8] and CCR7^+ ^naive and memory T lymphocytes have been detected in both normal and inflamed human tissues [9]. Previous studies have shown that Th1-polarised [10,11], CCR5^+ ^and CXCR3^+ ^lymphocytes are enriched in synovial inflammatory infiltrates and in synovial fluid (SF) lymphocytes from patients with adult rheumatoid arthritis (RA) [12,13] and juvenile idiopathic arthritis (JIA) [14-16]. CCR5 and CXCR3 ligands, namely RANTES (or CCL5) and macrophage inhibitory protein-1α (MIP-1α, or CCL3), and interferon-inducible protein-10 (IP-10, or CXCL10) and ITA-C (CXCL11), respectively, have also been detected in rheumatoid synovium [17].

Limited information is available on CCR7 expression in synovial lymphocytes from patients with chronic arthritis. Naive CD45RA^+ ^T cells with a CCR7 phenotype have been found to infiltrate the synovial tissue in patients with RA [16]. The CCR7 ligands CCL19 and CCL21 have been detected in endothelial cells and in the perivascular infiltrate in RA synovium, suggesting their potential involvement in lymphoid neogenesis that occurs in inflamed synovial tissue [18-20].

No information is so far available on the expression of CCR7 in memory T cells homing to the synovial microenvironment in relation to expression of the inflammatory chemokine receptors CCR5 and CXCR3.

In this study we therefore investigated the expression of CCR7, CCR5 and CXCR3 on SF and peripheral blood (PB) memory CD4^+ ^T cells from patients with JIA, chemotaxis of the latter cells to the ligands of these receptors, and the distribution of cells positive for CCR7, CCR5 and CXCR3 in the inflamed synovium.

## Methods

### Patients

Immunophenotypic and functional characterisation of freshly isolated PB and/or SF lymphocytes was performed in a total of 25 patients with JIA (14 female, 9 male) undergoing therapeutic arthrocentesis. According to ILAR Durban classification criteria [21], 15 patients had persistent oligoarticular JIA (pOJIA), 6 had extended oligoarticular JIA (eOJIA) (which means a total of five or more joints involved after the first 6 months of disease and therefore a polyarticular course) and 4 had rheumatoid factor (RF)-negative polyarticular JIA. Several clinical (number of active joints, number of joints with limited range of motion, and physician global assessment of overall disease activity) and laboratory parameters (erythrocyte sedimentation rate, C-reactive protein, white blood cell and platelet counts, and hemoglobin serum concentration) of disease activity were recorded, together with the ongoing treatment, at the time of the study.

Paired serum and SF samples from 28 additional patients with JIA (16 with pOJIA, 6 with eOJIA, 4 with RF negative polyarticular JIA, and 2 with systemic JIA) were tested for CCL21 concentrations. The clinical characteristics of patients with JIA and the ongoing treatment at the time of the study are reported in Tables 1 and 2. For each patient, SF was collected at the time of intra-articular steroid injection. Paired serum sample was obtained, with permission, on the occasion of concomitant routine venipuncture. Both SF and sera were stored at -80°C immediately after centrifugation. A previous steroid injection into the same joint in the previous 6 months was considered to be an exclusion criterion.

Peripheral blood and/or sera from 15 age-matched healthy subjects attending our clinic for routinary pre-operative examinations for minor surgical procedures were used as controls. Synovial tissue from six patients (two with pOJIA, one with eOJIA and three with RF-negative polyarticular JIA) was obtained, with permission, at the time of synoviectomy.

Samples were taken from patients and healthy controls, and stored after parental permission in accordance with the informed consent approved by the ethical committee of the 'G. Gaslini' Institute.

### Cell preparation and flow cytometry

PB and SF mononuclear cells (MNC) were isolated from heparinised blood and SF samples by Ficoll–Hypaque (Sigma, St Louis, MO, USA) density gradient centrifugation. Cells were washed, resuspended in complete medium (RPMI 1640 with L-glutamine, penicillin/streptomycin, nonessential amino acids and 10% fetal bovine serum; Sigma) and depleted of adherent cells by adherence to plastic for 1 h at 37°C in 5% CO_2_. To analyse the expression of CCR7 on CD4^+ ^memory T cells in SF and PB MNC, cells were triple-stained with CD45RO-TC (Caltag, Burlingame, CA, USA), CD4–FITC (BD Biosciences, San Jose, CA, USA) and anti-CCR7–PE (BD Pharmingen, San Diego, CA, USA) monoclonal antibodies (mAbs) and analysed by flow cytometry (CellQuest software and FACScan; BD Biosciences). CCR7 expression was evaluated by gating on the CD45RO^+^CD4^+ ^lymphocyte population. CD45RO^+ ^cells were purified from PB and SF MNC by negative selection with a CD45RA mAb (Caltag) and goat anti-mouse IgG-coated magnetic beads (Immunotech, Marseille, France), in accordance with the manufacturer's instructions. Recovered cells were 95% enriched for CD45RO^+ ^cells.

CCR5 or CXCR3 expression was investigated by three-colour staining of freshly isolated SF and PB CD45RO^+ ^cells with fluorescein isothiocyanate (FITC)-conjugated CD4 (BD Biosciences), anti-CCR7–phycoerythrin (PE) and anti-CCR5–CyChrome mAbs (BD Pharmingen) or CD4-TC (where TC stands for Tri-color), anti-CCR7–PE and anti-CXCR3–FITC (R&D System, Minneapolis, MN, USA), respectively, gating on the CD4^+^CCR7^+ ^and CD4^+^CCR7^- ^lymphocyte populations.

For interferon (IFN)-γ intracellular staining, freshly purified SF CD45RO^+ ^cells (10^6^) were incubated for 5 hours in the presence of phorbol 12-myristate 13-acetate (20 ng/ml; Sigma), the calcium ionophore A-23187 (250 ng/ml; Sigma) and brefeldin-A (5 μg/ml; Sigma). Cells were washed in phosphate-buffered saline (PBS) with 1% fetal calf serum (staining buffer) and surface stained with CD4–TC (Caltag) and anti-CCR7–PE (BD Pharmingen) mAbs for 30 min at 4°C in the dark. Cells were washed in staining buffer and fixed in 4% paraformaldehyde for 20 min at 4°C in the dark. Afterwards, the cells were washed twice with permeabilisation buffer (PBS containing 1% fetal calf serum and 0.1% saponin [Sigma]) and stained with FITC-conjugated mAbs against human IFN-γ (Caltag) for 30 min at 4°C in the dark. Cells were then washed in staining buffer and analysed by flow cytometry, gating on the CD4^+^CCR7^+ ^and CD4^+^CCR7^- ^lymphocyte subsets. Although stimulation with phorbol 12-myristate 13-acetate and calcium ionophore downregulates the intensity of CD4 and CCR7 expression, the proportion of cells positive for each marker was similar before and after stimulation.

Isotype matched, PE-, FITC-, TC- and CyChrome-conjugated mAbs of irrelevant specificity were tested as negative controls in all of the above experiments. The results of flow cytometry experiments were expressed as percentage positive cells or as mean fluorescence intensity; that is, the staining intensity of a test mAb minus that of an isotype-matched, irrelevant control mAb. The threshold for calculating the percentage positive cells was based on the maximum staining obtained with irrelevant isotype-matched mAb, used at the same concentration as the test mAb. Negative cells were defined such that less than 1% of cells stained positive with control mAbs. Cells labelled with test antibody that were brighter than those stained with isotypic control antibody were defined as positive. Mean fluorescence intensities of the isotype control and of test mAbs were used to evaluate whether the differences between the peaks of cells were statistically significant with respect to the control. The Kolmogorov–Smirnov test for the analysis of histograms was used, in accordance with the CellQuest software user's guide. Differences between paired PB and SF MNC of patients with JIA on the one hand, and PB MNC of healthy controls on the other, were evaluated by the Kruskal–Wallis analysis of variance (ANOVA) test and the Wilcoxon rank test.

### Chemotactic assays

Migration assays were performed in 24 transwell plates (pore size 5 μm, polycarbonate membrane; Costar, Cambridge, MA, USA). Freshly purified SF CD45RO^+ ^cells (5 × 10^5^) were dispensed in the upper chamber in 100 μl, and 600 μl of different chemokines at 100 ng/ml (R&D System) or medium alone was added to the lower chamber. Migration was performed in migration medium (RPMI 1640, 0.1% bovine serum albumin; Sigma). Plates were incubated for 2 hours at 37°C. After removal of the transwell inserts, cells from the lower compartments were collected. Furthermore, 0.5 ml of 5 mM EDTA was added to the lower chamber for 15 min at 37°C to detach adherent cells from the bottom of the wells. Detached cells were pooled with the previously collected cell suspensions and counted by staining with trypan blue. To evaluate the percentage of migrated CD4^+ ^lymphocytes, cell suspensions were double-stained with CD4–PE and CD3–FITC mAbs (BD Biosciences) before and after migration and analysed by flow cytometry. The percentage input was calculated as follows: 100 × (cells migrated to chemokine/total cell number). Differences between cells that migrated to a given chemokine and the same cells that migrated in medium alone were calculated with non-parametric Wilcoxon rank test.

### CCL21 serum and SF concentrations

Forty-three sera (15 from controls) and 28 SFs were tested for CCL21 by an enzyme-linked immunosorbent assay kit from R&D System (Minneapolis, USA), in accordance with the instructions of the manufacturer.

Serum levels of CCL21 were compared in three groups of patients (12 patients with JIA with a polyarticular course, 16 patients with JIA with an oligoarticular course and 15 healthy controls) with the use of the non-parametric Kruskal–Wallis ANOVA test. Correlations between all the variables considered were evaluated with the non-parametric Spearman rank test. Differences between paired serum and SF chemokine concentrations were evaluated by the Wilcoxon rank test.

### Immunohistochemical studies

Tissue specimens with sizes between 5 and 12 mm were treated for single and double immunohistochemical stainings with a standard technique as reported previously [22]. In brief, all specimens were fixed in 4% formalin for 24 hours, then dehydrated and embedded in paraffin. Sections 4 μm thick were layered on polylysine-coated slides. Slides were deparaffinised in xylene, and rehydrated in a descending sequence of ethanol concentrations (100–70%).

Three different immunohistochemical techniques, namely alkaline phosphatase–anti-alkaline phosphatase (APAAP) for CCR7, avidin–biotin complex for CD21, and indirect immunoperoxidase (CD3, CD4, CD45RO, CD20, CCR5, CXCR3, CCL19 and CCL21), were performed after 30 min of warming in an oven in citrate buffer, pH 6, with subsequent inhibition of endogenous peroxidase. For single staining, tissue sections were incubated overnight at 4°C with the anti-CCR7 murine mAb, clone 2H4 (Pharmingen). Incubation of tissue sections with anti-CCL21 goat antiserum (R&D), anti-CCR5, clone 2D7 (Pharmingen), anti-CXCR3, clone 1C6 (Pharmingen), anti-CD3 (Dako, Glostrup, Denmark), anti-CD4, clone 4B12 (Neomarkers, Fremont, CA, USA), anti-CD20, clone L26 (Dako) and anti-CD45RO, clone UCHL1 (Menarini, Firenze, Italy) and anti-CD31 clone JC70A (Dako) was performed overnight at 4°C.

Sections were subsequently reacted for 30 min at room temperature (20–25°C) with (1) anti-mouse Ig antibody conjugated to peroxidase-labelled dextran polymer (EnVision; Dako) for CD3, CD45RO, CCR5 and CCR7 stainings, (2) anti-goat secondary biotinylated antibody, followed by high-sensitivity streptavidin–horseradish peroxidase conjugate for CCL21 determination (Cell and Tissue Staining kit; R&D), and (3) APAAP-conjugated rabbit anti-mouse Ig (1:25 dilution; Dako) antibody for CCR7 determination. The chromogenic diaminobenzidine substrate (Dako) was applied for 10 min. All washings were performed by incubating the sections in PBS. For CCR7 determination the alkaline phosphatase reaction was performed with a medium containing Tris-HCl buffer pH 8.2, naphthol AS-TR salt (Sigma) and levamisole (Sigma), for 20 min at 98°C. Slides were counterstained with Mayer's haematoxylin. For double CCR7/CCL21 staining, the sections were subjected to peroxidase reaction with goat CCL21 and were washed three times in Tris-buffered saline. Subsequently, the APAAP technique (see above) was applied with the mouse CCR7 Ab (at room temperature, for 3 hours). The secondary reagents were applied for 30 min each. For CCR7 and CCL21, a reactive lymph node from a 10-year-old boy was considered as positive control. Reactions in the absence of primary antibody and with irrelevant antibodies of the same isotypes (anti-cytomegalovirus, clones DDG9 and CCH2; Dako) were performed as negative controls.

Slides were evaluated on two different occasions by two blinded observers (MG and AG) and an expert pathologist (CG). Each specimen was evaluated for the pattern of lymphocyte infiltration in three different categories: (1) aggregates of T cells (CD4) and B cells (CD20) with germinal centre (GC)-like reaction (presence of CD21-positive cells), (2) aggregates of T and B cells without GC-like reaction, and (3) diffuse lymphocytic infiltrate without lymphoid organisation [20]. For each sample a semiquantitative score for the overall degree of T lymphocyte infiltration (CD3) was used (range 0–3). For the assessment of chemokine receptor expression each sample was subjected to microscopical analysis of: (1) the lining layer and sublining zone of the superficial subintima [23]; (2) perivascular infiltrates of the sublining layer without lymphoid organisation; (3) aggregates of T and B cells. Because CCR7, CXCR3 and CCR5 can be expressed by several cell types (lymphocytes, dendritic cells, B cells and plasma cells) [18,24], only areas characterised by a clear lymphocyte infiltration (as defined by anti-CD3 and anti-CD4 positivity) were taken into consideration. The following semiquantitative global score was based on a visual inspection of four different high-power fields (40×) at each level: absent (-, no positive cells per high-power field), weakly positive (+, 1–10 positive cells per high-power field), moderately positive (++, 10–20 positive cells per high-power field), and strongly positive (+++, more than 20 positive cells per high-power field). The assignment of each sample to one of the above categories was based on the predominant pattern observed. Minor differences between the observers were resolved by mutual agreement. Intra observer and interobserver variability was less than 5%.

## Results

### Phenotypic and functional characterisation of CCR7^+ ^and CCR7^- ^CD4^+ ^memory T cells isolated from SF

Expression of CCR7 on CD4^+ ^memory T cells from the PB and SF of 10 patients with JIA was investigated by three-colour immunofluorescence analysis and compared with that detected on the same PB cell subset from eight age-matched healthy controls.

The heterogeneity test between the three subgroups was highly significant (Kruskal–Wallis ANOVA test, *P *= 0.0001). At *post hoc *analysis, in the PB from patients with JIA, the percentage of CCR7^+ ^cells in the CD4^+^CD45RO^+ ^subpopulation (median 65.5%, range 50–90%) was significantly lower than in PB from controls (median 76%, range 73–89%, *P *= 0.03, Mann–Whitney *U*-test). A further decrease in CCR7^+ ^cells was observed in memory CD4^+ ^cells isolated from SF (median 41.2%, range 12–59%) in comparison with paired PB.

Thus, even a variable proportion of SF memory CD4^+ ^T cells are positive for CCR7; this subpopulation is clearly enriched in CCR7^- ^cells in comparison with paired PB.

Next we investigated the expression of CCR5, CXCR3 and IFN-γ in SF CD4^+^CD45RO^+^CCR7^+ ^and CCR7^- ^cells from 10 consecutive patients and compared it with that detected in paired PB from 5 of these patients and in the PB of 5 healthy controls. To this end, purified CD45RO^+ ^cells were stained with anti-CD4, anti-CCR7 and respectively, anti-CCR5, CXCR3 or IFN-γ mAbs in three-colour immunofluorescence.

**Figure 1:**
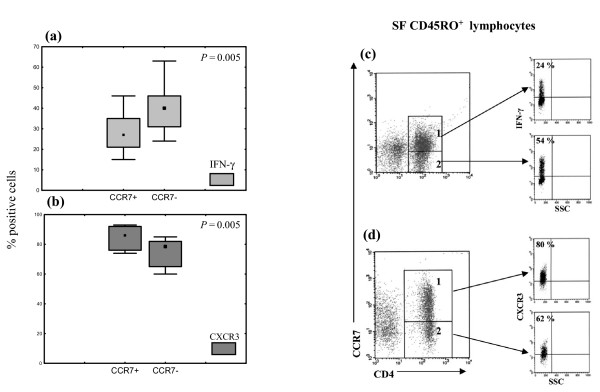
Expression of CXCR3 and interferon (IFN)-γ by (SF) CCR7^+ ^and CCR7^- ^memory CD4^+ ^cells from synovial fluid. IFN-γ expression was investigated by three-colour staining of freshly isolated SF CD45RO^+ ^cells with CD4–fluorescein isothiocyanate (FITC), anti-CCR7–phycoerythrin (PE) and anti-CCR5–CyChrome monoclonal antibodies (mAbs) or CD4–TC (where TC stands for Tri-color), anti-CCR7–PE and anti-IFN-γ mAbs, respectively; CXCR3 expression was investigated by triple staining with CD4–TC, anti-CCR7–PE and anti-CXCR3–FITC, as described in the Methods section. Subsequently, cytofluorimetric analysis was performed by gating on the CD4^+^CCR7^+ ^and CD4^+^CCR7^- ^lymphocyte subsets. Data are expressed as percentages of positive cells or/and mean fluorescence intensity. **(a, b) **Expression of IFN-γ (a) and CXCR3 (b) by SF CCR7^+ ^and CCR7^- ^memory CD4^+ ^cells from 10 patients with juvenile idiopathic arthritis (JIA). Boxes contain values falling between the 25th and 75th centiles; whiskers show lines that extend from the boxes represent the highest and lowest values for each subgroup. Differences between paired SF mononuclear cells were evaluated by the Wilcoxon rank test. **(c, d) **Dot plots show the cytofluorimetric analysis IFN-γ (c) and CXCR3 (d) expression by the gated CD4^+^CCR7^+ ^(gate 1) and CD4^+^CCR7^- ^(gate 2) cell populations in three representative patients with JIA.

In SF, CCR5^+ ^cells were found to be enriched in the CD4^+^CD45RO^+^CCR7^- ^lymphocyte subset (median 85%, range 74–99%) as compared to the CD4^+^CD45RO^+^CCR7^+ ^cell fraction (median 65%, range 46–84%, *P *= 0.005; Wilcoxon test; not shown). These data were in line with our previous observation of a higher expression of CCR7 in 'early' CD27^+ ^memory T cells and a prevalent CCR7^-^CCR5^+ ^phenotype in 'effector' CD27^- ^T cells in SF from patients with JIA [25]. The median percentage of CD4^+^, CD45RO^+^, IFN-γ positive cells was 27% (range 21–46%) for CCR7^+ ^cells and 40% (range 24–69%) for CCR7^- ^cells (*P *= 0.005) (Fig. 1a,c), as assessed by intracellular staining. Accordingly, the mean fluorescence intensity for IFN-γ was lower for CCR7^+ ^cells (median 136, range 84–184) than for paired CCR7^- ^CD4^+ ^memory T cells (median 190, range 127–307, *P *= 0.005).

CXCR3 was highly expressed on both CCR7^+ ^and CCR7^- ^subsets of SF memory CD4^+ ^T cells. However, in all patients with JIA, SF CCR7^+ ^memory CD4^+ ^T cells showed a higher expression of CXCR3 (median 86%, range 74–93%) than the CCR7^- ^counterpart (median 76%, range 62–85, *P *= 0.005) (Fig. 1b,d). In comparison with SF, PB of patients with JIA showed a lower expression of CXCR3 in both CCR7^+ ^(median 38.5%, range 24–55%) and CCR7^- ^(median 27%, range 17–40%) CD4^+ ^memory T cells. A similar expression was also found in circulating CCR7^+ ^(median 40%, range 32–55%) and CCR7^- ^(median 17%, range 13–45%) CD4^+ ^memory T cells from age-matched healthy controls.

Taken together, these results show that the CD4^+^CD45RO^+^CCR7^- ^subpopulation is enriched in 'effector' CCR5 and IFN-γ expressing cells, whereas the CD4^+^CD45RO^+^CCR7^+ ^subpopulation shows a lower expression of CCR5 and IFN-γ and a higher degree of coexpression with CXCR3.

### Different localisation of CCR7, CXCR3 and CCR5 positive cells in synovial tissue

We next addressed the following questions: (1) is CCR7 expressed in synovial tissue, (2) how does its expression correlate with the pattern of lymphocytic infiltration, and (3) how is CCR7 expression related to that of CXCR3 and CCR5, two Th1-associated chemokine receptors? To this end, synovial tissues obtained at synoviectomy from six patients with JIA were analysed for the expression of CCR7, CXCR3 and CCR5 in areas characterised by a clear lymphocyte infiltration (Table 3).

Different patterns in the synovial inflammatory infiltrate were observed in the individual patients (Table 3). One patient (no. 6) showed T and B cell aggregates with the presence of a GC reaction, as demonstrated by the presence of CD21^+ ^follicular dendritic cells [20]. In three patients (nos 3, 4 and 5) clusters of T and B cell aggregates in the absence of follicular dendritic cells were observed [20]. Two patients (nos 1 and 2) displayed diffuse lymphocytic infiltrates as perivascular aggregates in the sublining layer or scattered throughout the synovium up to the lining layer [20].

**Figure 2:**
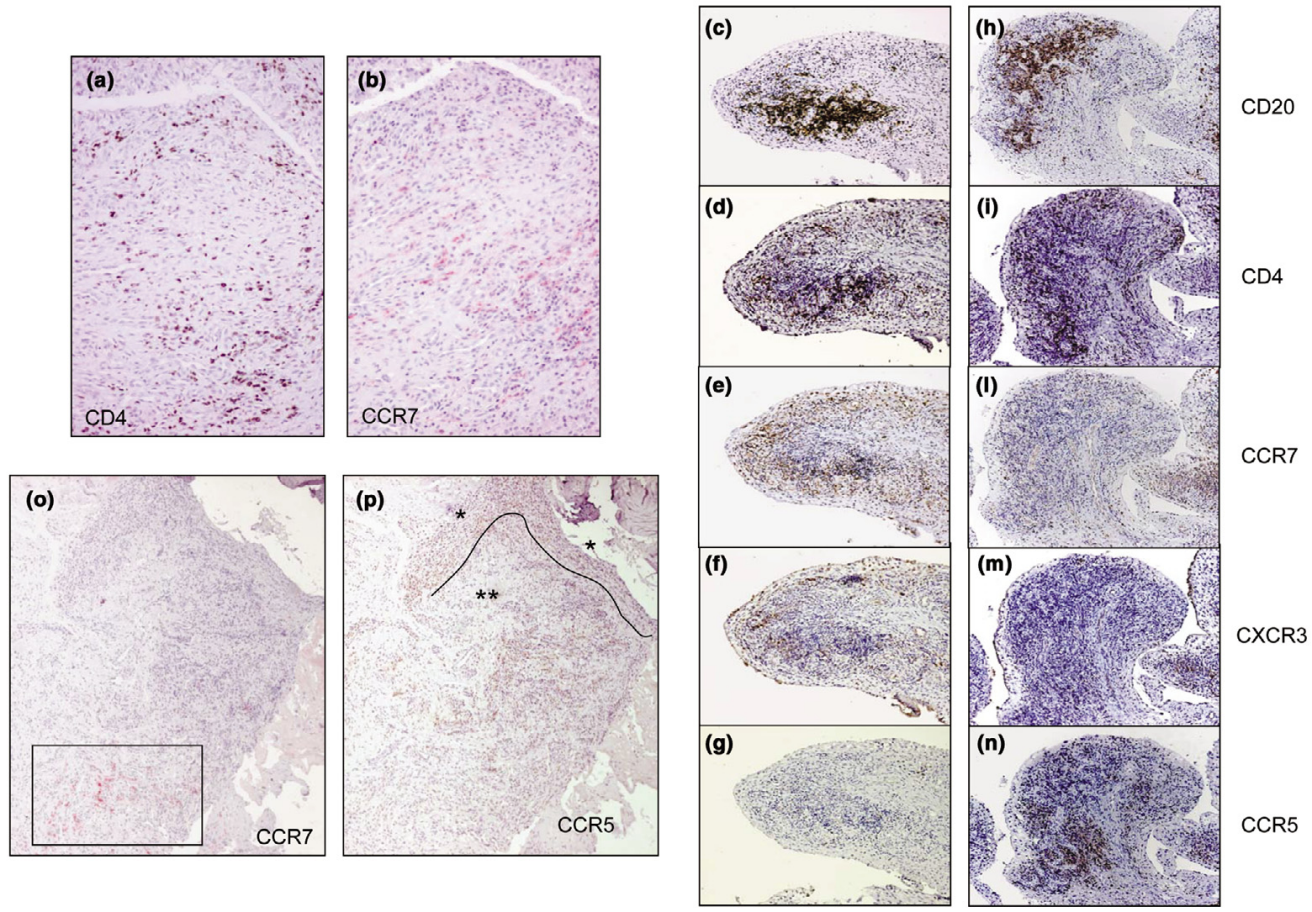
Expression of CD4, CD20, CD45RO, CCR7, CCR5 and CXCR3 in synovial tissue obtained from patients with juvenile idiopathic arthritis (JIA) after synoviectomy. **(a, b) **Presence of CCR7^+ ^cells (red) in the sublining layer of a synovial tissue characterised by a diffuse lymphocytic infiltrate (scattered CD4^+ ^cells, brown) from a 14-year-old girl with antinuclear antibody-positive (ANA^+^) oligoarticular JIA (no. 1, Table 3) (Magnification × 20). (**c–n) **Serial stainings with CD20, CD4, CCR7, CXCR3 and CCR5 monoclonal antibody in synovial tissue from a 12-year-old girl with ANA^+ ^oligoarticular JIA (no. 4, Table 3). The distribution of cells positive for CCR7, CCR5 and CXCR3 is shown in two different areas containing T and B cell aggregates (magnification × 10). **(o, p) **Different expression of CCR7 (red) and CCR5 (brown) in synovial membrane infiltrate (patient no. 1, Table 3) (magnification × 4.5). CCR7^+ ^cells are observed exclusively in the perivascular lymphocytic infiltrate of the deep sublining layer (open rectangle). Conversely, CCR5-positive cells are prevalently observed at the level of the lining layer and superficial subintima (*) and in the perivascular infiltrates of the sublining layer (**).

CCR7-positive cells were detected both in cases showing a diffuse lymphocytic infiltrate (Fig. 2a,b) and in those displaying a more organised lymphoid structure (Fig. 2c–e). In the former, CCR7 expression was detected mostly in the perivascular lymphocytic infiltrates of the sublining layer (Fig. 2b,o) and, only occasionally, in scattered cells in the sublining zone of the superficial subintima (see also Fig. 4c below). In the latter, CCR7-positive cells were localised inside and around lymphoid aggregates (Fig. 2e).

Because naive CD45RA^+ ^T cells with a CCR7^+ ^phenotype had also previously been found to infiltrate the synovial tissue from patients with RA [19], serial sections were stained with CCR7 and CD45RO antibodies. A clear positivity for CCR7 was detected in lymphocytic infiltrates staining heavily for CD45RO (not shown).

CXCR3 was abundantly expressed in all lymphocyte-infiltrated areas examined (Table 3). In fact, CXCR3-positive cells were detected in lymphoid aggregates (Fig. 2f) and in perivascular infiltrates of sublining layer (Table 3). In many areas, CXCR3 and CCR7 displayed a similar pattern of tissue distribution, especially at the level of lymphocyte aggregates (Fig. 2e,f).

Conversely, CCR5-positive cells were detected mainly in the lining layer and in the sublining zone of the superficial subintima and, to a smaller extent, in the perivascular infiltrates of the sublining layer (Fig. 2p) and in the T and B cell aggregates (Fig. 2n) (Table 3).

Altogether, even if a certain degree of co-localisation of the two chemokine receptors was found (Table 3), CCR5 positive cells showed a substantially different tissue distribution from that of CCR7, either in T and B cell aggregates (Fig. 2e,g,l,n) or in diffuse lymphocytic infiltrates (Fig. 2o,p). Conversely, a variable degree of co-localisation was found for CCR5 and CXCR3 at the level of the sublining and lining layer (Table 3).

### Chemotaxis of SF CD4^+ ^memory T cells to inflammatory and homeostatic chemokines

In further experiments, chemotaxis of freshly isolated SF memory T cells in response to CCR7, CCR5 and CXCR3 ligands was investigated, and migrated CD4^+^CD45RO^+ ^T cells were detected by flow cytometry.

Chemotactic assays were performed with SF CD45RO^+ ^cells isolated from eight patients with JIA (five with pOJIA, three with eOJIA) and tested in the presence or absence of two inflammatory chemokines that bind to CCR5 (CCL3) and CXCR3 (CXCL11), respectively, and of homeostatic chemokines binding to CCR7 (CCL21 and CCL19).

**Figure 3:**
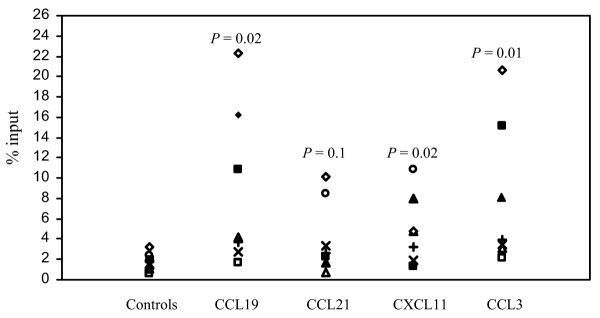
Chemotactic activity of CD4 memory T cells from the synovial fluid of eight patients with juvenile idiopathic arthritis to inflammatory (CXCL11 and CCL3) and homeostatic (CCL19, CCL21) chemokines. Results are expressed as the percentage of migrated cells in the total cell input (see also the Methods section).

CD4^+^CD45RO^+ ^T cells migrated significantly to both CCL3 and CXCL11 (*P *= 0.02 for both chemokines). Similar responses were observed when CCL19 was tested (*P *= 0.02). Chemotaxis of CD4^+ ^memory T cells to CCL21 approached but did not reach statistical significance (*P *= 0.1) (Fig. 3). The latter finding might be related to the limited number of the samples tested.

In the patients studied, the variability of chemotaxis of SF CD4^+ ^memory T cells did not show any significant correlation with disease form, degree of disease activity and treatment at the moment of sampling.

### Expression of CCL21 in SF and synovial tissue

To gain further insight into the relevance of the interactions between CCR7 and its ligand CCL21 *in vivo*, sera and SF CCL21 concentrations were tested in 28 consecutive patients with JIA and in 15 healthy controls.

**Figure 4:**
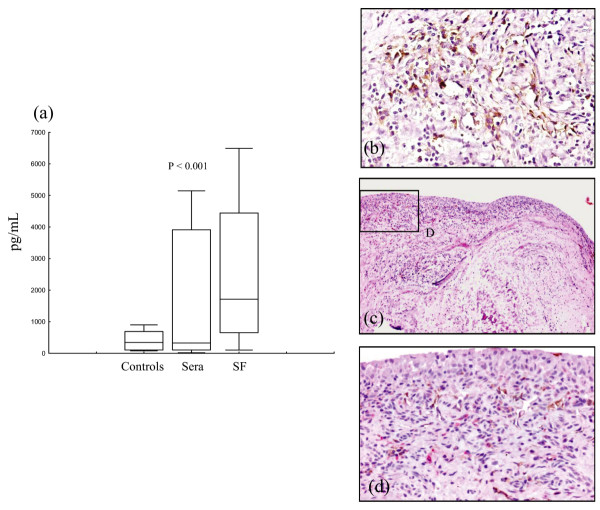
Expression of CCL21 in synovial fluid and tissue. **(a) **CCL21 concentrations in sera from 15 age-matched healthy controls, paired sera (Sera) and synovial fluids (SF) from 28 patients with juvenile idiopathic arthritis (JIA). Lines represent median values. Boxes contain values falling between the 25th and 75th centiles; whiskers show lines that extend from the boxes represent the highest and lowest values for each subgroup. The heterogeneity test among the three subgroups was highly significant (Kruskal–Wallis analysis of variance test, *P *= 0.0045). At *post hoc *analysis, differences between paired sera and SF were evaluated by the Wilcoxon rank test. Difference between JIA sera and healthy controls were evaluated by the Mann–Whitney *U*-test. **(b) **Expression of CCL21 in perivascular aggregates and vascular endothelium in synovial tissue with diffuse lymphocytic infiltration from 10-year-old girl with persistent oligoarticular JIA. **(c, d) **Double staining with anti-CCR7 (red) and anti-CCL21 (brown) monoclonal antibodies at different magnifications (×10 and ×40, respectively) shows CCL21 expression by endothelial cells of vessels located in the sublining zone of the superficial subintima.

The heterogeneity test between the three subgroups was highly significant (Kruskal–Wallis ANOVA test, *P *= 0.0045). Concentrations of CCL21 were significantly higher in SF (median 1769.5 pg/ml, range 110–25,556 pg/l) than in paired sera from patients with JIA (median 268 pg/ml, range 57.6–5146.9 pg/ml, *P *< 0.0001; Wilcoxon test; Fig. 4a).

A strong correlation was found between paired serum and SF CCL21 concentrations (*r *= 0.91, *P *= 0.001; Spearman's test).

No significant difference was observed in CCL21 serum concentrations between patients with JIA with oligoarticular course (median 229.2 pg/ml, range 67–3948 pg/ml), patients with JIA with polyarticular course (median 378 pg/ml, range 65–5146 pg/ml) and age-matched healthy controls (median 282.2 pg/ml, range 76–2349 pg/ml, *P *= 0.3; Kruskal–Wallis ANOVA test). Similarly, no significant difference was found in SF CCL21 concentrations between patients with JIA with an oligoarticular course and patients with a polyarticular course (*P *= 0.52; Mann–Whitney *U*-test).

Finally, no significant correlation was found between CCL21 serum concentrations and several clinical and laboratory parameters of disease activity in patients with JIA (see the Methods section; not shown).

The expression of CCL21 was also analysed in synovial tissues by immunohistochemistry. CCL21 was detected in all specimens. In the samples characterised by lymphoid organisation, CCL21 staining was observed in the perivascular lymphocytic aggregates and in the vascular endothelium within follicular structures, a pattern reminiscent of that observed on staining for CCR7 [19]. A similar pattern was detected in tissues showing a diffuse lymphocytic infiltration (Fig. 4b). Moreover, a clear-cut expression of CCL21 was also observed in flat wall vessels of the superficial subintima of the sublining layer (Fig. 4c,d) [23].

## Discussion

In this study we have investigated the role of CCR7 in the recruitment of CD4^+ ^memory T cells into the inflamed joints of patients with JIA, and attempted the functional and anatomical dissection of these cells according to their expression of CCR7, CXCR3, CCR5 and IFN-γ. We detected two populations of SF CD4^+ ^memory T cells: the CD4^+^CD45RO^+^CCR7^- ^subset, which was enriched in 'effector' CCR5 and IFN-γ positive cells, and the CD4^+^CD45RO^+^CCR7^+ ^subset, which was less well represented and showed higher CXCR3 coexpression. SF CD4^+ ^memory T cells displayed chemotactic activity to both inflammatory and homeostatic chemokines representing the physiological ligands of these receptors.

Of the three chemokine receptors studied, CXCR3 proved to be the most widely expressed in synovial tissue, with a clear distribution both in lymphoid aggregates and in perivascular infiltrates of sublining layer and in the lining layer.

Conversely, CCR7-positive and CCR5-positive cells in the synovial tissue displayed a different distribution, showing an even higher differentiation in their expression in respect to SF. In fact, CCR7^+ ^cells were detected in synovial tissues irrespective of the pattern of lymphoid organisation and were localised mainly in lymphoid aggregates and in perivascular infiltrates of the sublining layer. Notably, CCL21, the CCR7 ligand, was found in the SF as well as in perivascular lymphocytic aggregates and in the vascular endothelium of follicular structures.

In contrast, CCR5^+ ^cells were detected mainly in the lining layer and in the sublining zone of the superficial subintima and, to a smaller extent, in the perivascular infiltrates of sublining layer and in the T and B cell aggregates.

These findings in synovial tissue are in line with the results of the phenotypic characterisation of SF CCR7^+ ^and CCR7^- ^memory CD4^+ ^T cells performed in the present study and with previous observations showing a variable degree of coexpression of CXCR3 and CCR5 on T cells isolated from inflamed tissues [14,26,27].

To our knowledge, this is the first demonstration of a different anatomical localisation of cells positive for CCR7, CCR5 and CXCR3 infiltrating the inflamed synovium; this finding may have functional implications for the intra-tissue migration of T cells.

During the past decade several studies have focused on the capacity of memory T cells to differentiate in the context of inflamed tissues. Many of these studies used a member of the tumour necrosis factor receptor family, CD27, to distinguish recently activated CD27^+ ^from 'effector' CD27^- ^memory CD4^+ ^T cells [28]. Notably, a clear enrichment of the latter subpopulation has been found in SF of patients with RA and JIA [29,30]. In a recent study we showed that CD27^+ ^memory T cells in SF of patients with JIA expressed CCR7 more highly than CCR5, whereas CD27^- ^T cells displayed a prevalent CCR7^- ^CCR5^+ ^phenotype [25]. Notably, the immunohistochemical characterisation of rheumatoid synovial tissue in adult RA has shown a prevalent localisation of CD4^+^CD27^+ ^T cells in the perivascular lymphocytic aggregates, with a relative increase in CD27^- ^T cells in diffuse lymphocytic infiltrates [31].

Thus, it is conceivable that the functional and phenotypic characterisation of CCR7^+ ^and CCR7^- ^memory CD4^+ ^T cells and the different tissue distribution between CCR7 and CCR5 found in the present study might reflect the same behaviour already observed for CD27^+ ^and CD27^- ^memory T cells, yielding more insight into the migratory properties of memory T cells into and within the synovial tissue.

The partial overlap of CCL21 and CCR7 expression in the inflamed synovium might suggest that the CCR7/CCL21 system, probably in synergy with CXCR3 and its ligands, is involved in the recruitment of memory T cells, as already shown for naive T cells [19]. However, the possibility cannot be ruled out that CCR7 expression in CD4^+ ^memory T cells isolated from SF was upregulated after the reactivation of these cells at the site of inflammation [32].

CCL21, together with other homeostatic chemokines such as CXCL13, has been shown to have a fundamental function in the development of secondary lymphoid organs by interacting with CCR7 [4,33]. Mice whose CCR7 or CCL21 genes have been knocked out exhibit marked deficiencies in the structural and cellular composition of lymph nodes [34].

A sequence of events similar to that taking place in lymph node organogenesis is supposed to be involved in the development of organised lymphoid structures in inflamed tissues, such as the rheumatoid synovium [18-20]. Indeed, up to 20% of synovial tissue biopsies from patients with RA show the typical features of the GC reaction. Other patients show aggregates of T and B cells in the absence of an evident follicular organisation [20], whereas in more than 50% of synovial tissue samples from RA [20] and a considerable proportion of patients with JIA (M Gattorno, unpublished data), diffuse T and B lymphocytic infiltrates in the absence of aggregates or follicular structures are observed. Interestingly, in the individual patients with RA, the pattern of lymphocytic infiltration was found to persist unaltered over time, and showed similar features in all biopsies taken from different joints at the same type [20].

In our study, both CCR7 and its ligand CCL21 were found to be abundantly expressed in synovial biopsies, irrespective of the pattern of lymphoid infiltration. These observations support the hypothesis that CCR7 and its ligands have a direct function in the recruitment of memory T cells to the inflamed synovium, one that is independent of their ability to organise in lymphoid structures.

In this respect, the recent demonstration of different regulation of CCL21 in lymphoid and non-lymphoid tissues is noteworthy. Lymphotoxin-α directs the formation of lymph nodes and Peyer's patches through the induction of adhesion molecules and the production of chemokines, including CCL21, by the mesenchymal organiser cells during the early developmental steps [4,35]. Lymphotoxin-α-deficient mice show a marked impairment of lymphoid organisation in secondary lymphoid organs, but normal recruitment of naive and memory T cells to peripheral inflamed tissue through the CCR7/CCL21 system [36]. CCL21 and CCR7 might therefore either regulate lymphoid neogenesis by a lymphotoxin-dependent mechanism or recruit T cells to the inflamed tissues by a lymphotoxin-independent mechanism.

Taken together, our findings suggest that CCR7^+ ^memory T cells can be directly recruited, with the possible contribution of other chemokines such as CXCR3 ligands, to the synovium, where they undergo further differentiation leading to the downregulation of CCR7 from the cell surface and the concomitant upregulation of CCR5. This differentiation might be driven either by antigen-dependent or antigen-independent mechanisms. In fact, cytokines produced in the synovial microenvironment (namely interleukin-7 and interleukin-15) might allow the proliferation, expansion and differentiation of CCR7^+ ^memory T cells into effector cells, marked by the downregulation of CCR7, the upregulation of CCR5 and the production of IFN-γ [37]. In this model, CCR5 could represent the major chemokine receptor used for CD4^+ ^memory T cell locomotion within the inflamed tissue, according to a step-by-step navigation model through different chemoattractant gradients [38]. In contrast, the enrichment of CCR7^- ^CCR5^+ ^cells infiltrating the lining and sublining layer could be also related to the presence of other relevant effector cells, such as the granzyme B^+ ^cytotoxic cells [39].

## Conclusion

The present study delineates a coordinated pattern of expression of homeostatic and inflammatory chemokines in the inflamed synovium, with potential implications for the mechanisms regulating the intra-tissue migration and local differentiation of inflammatory cells.

## Abbreviations

ANOVA = analysis of variance; APAAP = alkaline phosphatase–anti-alkaline phosphatase; eOJIA = extended oligoarticular JIA; FITC = fluorescein isothiocyanate; GC = germinal centre; IFN = interferon; JIA = juvenile idiopathic arthritis; mAb = monoclonal antibody; MNC = mononuclear cells; PB = peripheral blood; PBS = phosphate-buffered saline; pOJIA = persistent oligoarticular JIA; RA = rheumatoid arthritis; RF = rheumatoid factor; SF = synovial fluid; TC = Tri color.

## Competing interests

The author(s) declare that they have no competing interests.

## Authors' contributions

MG conceived and coordinated the study, performed patients' selection and wrote the manuscript. IP, AM and VP participated in the study design and helped to draft the manuscript. IP, FM, SC and FF performed the cytofluorimetric analysis and chemotaxis studies. AU performed the enzyme-linked immunosorbent assay for CCL21 determination in sera and SF, and helped to draft the manuscript. AG, AF and CG performed the immunohistochemical analysis of synovial tissue. All authors read and approved the final manuscript.

## Figures and Tables

**Table 1:** Clinical and laboratory features of patients with juvenile idiopathic arthritis at the time of phenotypic and functional studies of peripheral blood and synovial fluid lymphocytes

Course	No. of patients	Age (years)	Disease duration (years)	No. of joints with active/limited range of motion	PGI	ESR (mm/h)	Treatment; *n*
Polyarticular	10	10.9 (3.3–16.1)	3.2 (0.5–11.2)	6 (1–16)/12.2 (1–29)	8.3 (6–10)	65 (23–131)	NSAID, MTX; 7
							NSAID, CS, MTX; 2
							NSAID alone; 1
Oligoarticular	15	8.5 (3–17.9)	2.4 (0.3–14)	1.3 (1–3)/1.5 (1–3)	6.3 (5–10)	26.5 (7–90)	NSAID alone; 11
							NSAID, MTX; 2
							Nil; 2

Results are means (ranges in parentheses).

CS, corticosteroids; ESR, erythrocyte sedimentation rate; MTX, methotrexate; NSAID, non-steroidal anti-inflammatory drugs; PGI, physician global index.

**Table 2:** Clinical and laboratory features of patients with juvenile idiopathic arthritis at the time of determination of CCL21 in sera and synovial fluid

Course	No. of patients	Age (years)	Disease duration (years)	No. of joints with active/limited range of motion	PGI	ESR (mm/h)	Treatment; *n*
Polyarticular	12	11.5 (4.3–14.3)	3.4 (0.5–1.2)	5.3 (1–13)/18.3 (1–22)	7.8 (5–10)	78 (24–137)	NSAID, MTX; 8
							NSAID, CS, MTX; 2
							NSAID, CS; 1
							NSAID alone; 1
Oligoarticular	16	7.5 (2.1–15.9)	2.4 (0.3–14)	1.8 (1–4)/1.9 (1–4)	6.9 (5–10)	31.5 (5–65)	NSAID alone; 11
							NSAID, MTX; 2
							Nil; 3

Results are means (ranges in parentheses). See also 'Patients' in the Methods section.

CS, corticosteroids; ESR, erythrocyte sedimentation rate; MTX, methotrexate; NSAID, non-steroidal anti-inflammatory drugs; PGI, physician global index.

**Table 3:** Distribution of chemokine receptors of synovial tissues from patients with juvenile idiopathic arthritis

Patient no.	Age (year)	Form	Pattern	CD3	CCR7			CXCR3			CCR5		
					Lining	Sublining	Aggregates	Lining	Sublining	Aggregates	Lining	Sublining	Aggregates
1	14	Oligo per.	Diffuse	+++	+	+++	NP	+++	+++	NP	+++	++	NP
2	19	Poly RF-	Diffuse	+	-	++	NP	+	+++	NP	+	-	NP
3	10	Oligo per.	T-B	++	-	+	++	+	++	++	++	+	-
4	12	Oligo ext.	T-B	+++	++	+++	+++	++	+++	+++	++	+	++
5	7	Poly RF-	T-B	++	-	-	+	++	+++	+++	+++	+	+
6	15	Poly RF-	GC	+++	-	+	++	+++	+++	+++	+++	+	-

Oligo per., persistent oligoarticular; oligo ext., extended oligoarticular; Poly RF-, polyarticular rheumatoid factor-negative; NP, not present; T-B, aggregates of T and B cells; GC, T and B cell aggregates with germinal centre (GC)-like reaction. Scoring: -, absence of positive cells in high-power field; +, 1–10 positive cells per high-power field; ++, 10–20 positive cells per high-power field; +++, more than 20 positive cells per high-power field (see also the Methods section). Lining, lining layer and superficial subintima; sublining, perivascular infiltrates in sublining layer; aggregates, T and B cell aggregates with or without GC-like reaction. All evaluations were performed in areas characterised by a clear lymphocyte (anti-CD3 and anti-CD4 positive cells) infiltration (see also the Methods section and Fig. 3).
